# *N*-(2-Methyl­phen­yl)maleamic acid

**DOI:** 10.1107/S160053681002012X

**Published:** 2010-06-05

**Authors:** B. Thimme Gowda, Miroslav Tokarčík, K. Shakuntala, Jozef Kožíšek, Hartmut Fuess

**Affiliations:** a Department of Chemistry, Mangalore University, Mangalagangotri 574 199, Mangalore, India; b Faculty of Chemical and Food Technology, Slovak Technical University, Radlinského 9, SK-812 37 Bratislava, Slovak Republic; c Institute of Materials Science, Darmstadt University of Technology, Petersenstrasse 23, D-64287 Darmstadt, Germany

## Abstract

In the title compound, C_11_H_11_NO_3_, the conformation of the N—H bond is *anti* to the C=O bond in the amide segment, while it is *syn* to the *ortho*-methyl group in the phenyl ring. In the maleamic acid unit, the amide C=O bond is *anti* to the adjacent C—H bond, while the carboxyl C=O bond is *syn* to the adjacent C—H bond. The C=O and O—H bonds of the acid group are in the relatively rare *anti* position to each other. This is an obvious consequence of the intra­molecular O—H⋯O hydrogen bond donated to the amide carbonyl group. The *ortho*-substituted phenyl ring makes a dihedral angle of 12.7 (1)° with the mean plane of the maleamic acid unit. In the crystal structure, inter­molecular N—H⋯O hydrogen bonds link the mol­ecules into zigzag chains parallel to [001]. These chains are further linked into sheet by weak π–π inter­actions [centroid–centroid distance = 3.425 (2) Å].

## Related literature

For studies on the effect of ring- and side-chain substitutions on the crystal structures of amides, see: Gowda *et al.* (2009**a* ▶,*b* ▶,c* ▶); Prasad *et al.* (2002 ▶). For the modes of inter­linking carb­oxy­lic acids by hydrogen bonds, see: Jagannathan *et al.* (1994 ▶); Leiserowitz (1976 ▶).
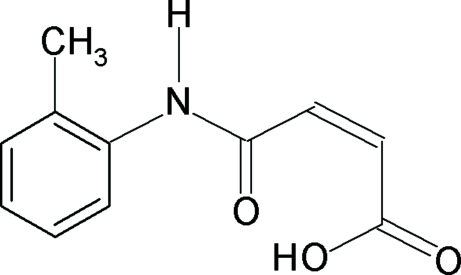


## Experimental

### 

#### Crystal data

C_11_H_11_NO_3_
*M* *_r_* = 205.21
Monoclinic, 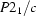

*a* = 7.3942 (3) Å
*b* = 11.5898 (4) Å
*c* = 12.9903 (3) Å
β = 114.534 (2)°
*V* = 1012.72 (5) Å^3^
*Z* = 4
Mo *K*α radiation
μ = 0.10 mm^−1^
*T* = 295 K
0.58 × 0.42 × 0.42 mm

#### Data collection

Oxford Diffraction Gemini R CCD diffractometer
Absorption correction: analytical (*CrysAlis PRO*; Oxford Diffraction, 2009 ▶) *T*_min_ = 0.922, *T*_max_ = 0.962
15644 measured reflections
1776 independent reflections
1453 reflections with *I* > 2σ(*I*)
*R*_int_ = 0.027

#### Refinement

*R*[*F* ^2^ > 2σ(*F* ^2^)] = 0.034
*wR*(*F* ^2^) = 0.098
*S* = 1.07
1776 reflections
137 parameters
H-atom parameters constrained
Δρ_max_ = 0.17 e Å^−3^
Δρ_min_ = −0.13 e Å^−3^

### 

Data collection: *CrysAlis PRO* (Oxford Diffraction, 2009 ▶); cell refinement: *CrysAlis PRO*; data reduction: *CrysAlis PRO*; program(s) used to solve structure: *SHELXS97* (Sheldrick, 2008 ▶); program(s) used to refine structure: *SHELXL97* (Sheldrick, 2008 ▶); molecular graphics: *ORTEP-3* (Farrugia, 1997 ▶) and *DIAMOND* (Bran­denburg, 2002 ▶); software used to prepare material for publication: *SHELXL97*, *PLATON* (Spek, 2009 ▶) and *WinGX* (Farrugia, 1999 ▶).

## Supplementary Material

Crystal structure: contains datablocks I, global. DOI: 10.1107/S160053681002012X/dn2569sup1.cif

Structure factors: contains datablocks I. DOI: 10.1107/S160053681002012X/dn2569Isup2.hkl

Additional supplementary materials:  crystallographic information; 3D view; checkCIF report

## Figures and Tables

**Table 1:** Hydrogen-bond geometry (Å, °)

*D*—H⋯*A*	*D*—H	H⋯*A*	*D*⋯*A*	*D*—H⋯*A*
N1—H1*N*⋯O3^i^	0.86	2.22	3.0665 (14)	167
O2—H2*A*⋯O1	0.92	1.56	2.4822 (13)	178

Symmetry code: (i) 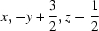.

**Fig. 1.**
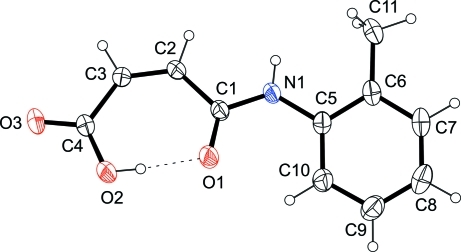
Molecular structure of (I) showing the atom labelling scheme. Displacement ellipsoids are drawn at the 30% probability level and H atoms are represented as small spheres of arbitrary radii.

**Fig. 2.**
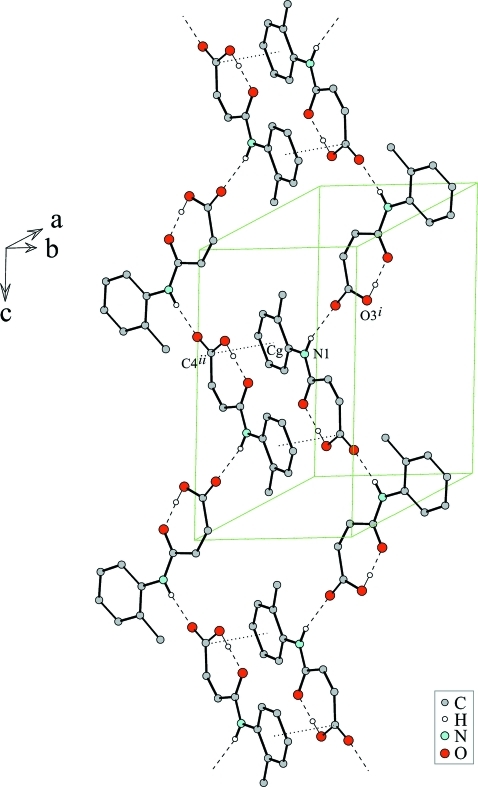
Part of the crystal structure of (I) showing the zigzag chains generated by N—H···O hydrogen bonds and extending parallel to the c axis. The chains are weakly coupled by π-π interaction between the phenyl rings and maleamic acid groups. The dashed lines depict the hydrogen bonds, the dotted line depicts the short contact Cg···C4ii. H atoms not involved in hydrogen bonding have been omitted. [Symmetry codes (i): x, -y + 3/2, z - 1/2; (ii) -x,-y + 1,-z + 1].

## supplementary crystallographic information

### Comment

The amide moiety is an important constituent of many biologically significant compounds. As a part of studying the effect of ring and side chain substitutions on the crystal structures of this class of compounds (Gowda *et al.*, 2009*a,b,c*; Prasad *et al.*, 2002), the crystal structure of *N*-(2-methylphenyl)-maleamic acid (I) has been determined (Fig. 1). The conformations of the N—H and the C=O bonds in the amide segment are *anti* to each other. But the conformation of the N—H bond is *syn* to the *ortho*-methyl group in the phenyl ring. In the maleamic acid moiety, the amide C=O bond is *anti* to the adjacent C—H bond, while the carboxyl C=O bond is *syn* to the adjacent C—H bond. The observed rare *anti* conformation of the C=O and O—H bonds of the acid group is similar to that obsrved in *N*-(2,6-dimethylphenyl)-maleamic acid (Gowda *et al.*, 2009*a*), *N*-(3,4-dimethylphenyl)-maleamic acid (Gowda *et al.*, 2009*b*) and *N*-(2,4,6-trimethylphenyl)- maleamic acid (Gowda *et al.*, 2009*c*).

The *ortho*-substituted phenyl ring makes a dihedral angle of 12.7 (1)° with the mean plane of the maleamic acid moiety (atoms C1, C2, C3, C4, N1, O1, O2 and O3). The orientation of the central amide group –NHOC– with respect to the phenyl ring is partially affected by the intramolecular hydrogen bond C10—H10···O1(amide) and is given by the torsion angle C10—C5—N1—C1 = -17.3 (2)°. Short intramolecular hydrogen bond O—H···O (Table 1) is important characteristic of the maleamic acid moiety. The C2–C3 bond length of 1.330 (2)Å clearly indicates the double bond character. In the crystal structure, the intermolecular N–H···O hydrogen bonds, having the amide N1 atom as donor and carbonyl O3 atom of the carboxyl group as acceptor, link the molecules into zigzag chains running along the [0 0 1] direction. Due to weak π-π interaction between the phenyl and maleamic acid moieties the chains are assembled to form sheets parallel to the *bc*-plane. One mode of the chain coupling is shown in Fig. 2 as a short contact between the phenyl ring centroid *Cg* and the C4 atom of the carboxylic group at (-*x*,-*y* + 1,-*z* + 1).

The various modes of interlinking carboxylic acids by hydrogen bonds is described elsewhere (Leiserowitz, 1976). The packing of molecules involving dimeric hydrogen bonded association of each carboxyl group with a centrosymmetrically related neighbor has also been observed (Jagannathan *et al.*, 1994).

### Experimental

The solution of maleic anhydride (0.025 mol) in toluene (25 ml) was treated dropwise with the solution of 2-methylaniline (0.025 mol) also in toluene (20 ml) with constant stirring. The resulting mixture was stirred for about 30 min and set aside for an additional 30 min at room temperature for the completion of reaction. The mixture was then treated with dilute hydrochloric acid to remove the unreacted 2-methylaniline. The resultant solid *N*-(2-methylphenyl)maleamic acid was filtered under suction and washed thoroughly with water to remove the unreacted maleic anhydride and maleic acid. It was recrystallized to constant melting point from ethanol. The purity of the compound was checked by elemental analysis and characterized by its infrared spectra. The single crystals used in X-ray diffraction studies were grown in an ethanol solution by slow evaporation at room temperature.

### Refinement

All H atoms attached to C atoms, N atom and O atom were fixed geometrically and treated as riding with C—H = 0.96 Å (methyl) or 0.93 Å (aromatic), N—H = 0.86 Å and O—H= 0.92Å with *U*_iso_(H) = 1.2*U*_eq_(C_aromatic_, N) or *U*_iso_(H) = 1.5*U*_eq_(C_methyl_, O).

### Crystal data

**Table:**

C_11_H_11_NO_3_	*F*(000) = 432
*M**_r_* = 205.21	*D*_x_ = 1.346 Mg m^−^^3^
Monoclinic, *P*2_1_/*c*	Mo *K*α radiation, λ = 0.71073 Å
Hall symbol: -P 2ybc	Cell parameters from 8927 reflections
*a* = 7.3942 (3) Å	θ = 2.5–29.5°
*b* = 11.5898 (4) Å	µ = 0.10 mm^−^^1^
*c* = 12.9903 (3) Å	*T* = 295 K
β = 114.534 (2)°	Prism, colourless
*V* = 1012.72 (5) Å^3^	0.58 × 0.42 × 0.42 mm
*Z* = 4	

### Data collection

**Table:**

Oxford Diffraction Gemini R CCD diffractometer	1776 independent reflections
graphite	1453 reflections with *I* > 2σ(*I*)
Detector resolution: 10.434 pixels mm^-1^	*R*_int_ = 0.027
ω scans	θ_max_ = 25°, θ_min_ = 2.5°
Absorption correction: analytical (*CrysAlis PRO*; Oxford Diffraction, 2009)	*h* = −8→8
*T*_min_ = 0.922, *T*_max_ = 0.962	*k* = −13→13
15644 measured reflections	*l* = −15→15

### Refinement

**Table:**

Refinement on *F*^2^	Primary atom site location: structure-invariant direct methods
Least-squares matrix: full	Secondary atom site location: difference Fourier map
*R*[*F*^2^ > 2σ(*F*^2^)] = 0.034	Hydrogen site location: inferred from neighbouring sites
*wR*(*F*^2^) = 0.098	H-atom parameters constrained
*S* = 1.07	*w* = 1/[σ^2^(*F*_o_^2^) + (0.056*P*)^2^ + 0.0996*P*] where *P* = (*F*_o_^2^ + 2*F*_c_^2^)/3
1776 reflections	(Δ/σ)_max_ < 0.001
137 parameters	Δρ_max_ = 0.17 e Å^−^^3^
0 restraints	Δρ_min_ = −0.13 e Å^−^^3^

### Special details

**Table:**

Geometry. All e.s.d.'s (except the e.s.d. in the dihedral angle between two l.s. planes) are estimated using the full covariance matrix. The cell e.s.d.'s are taken into account individually in the estimation of e.s.d.'s in distances, angles and torsion angles; correlations between e.s.d.'s in cell parameters are only used when they are defined by crystal symmetry. An approximate (isotropic) treatment of cell e.s.d.'s is used for estimating e.s.d.'s involving l.s. planes.
Refinement. Refinement of *F*^2^ against ALL reflections. The weighted *R*-factor *wR* and goodness of fit *S* are based on *F*^2^, conventional *R*-factors *R* are based on *F*, with *F* set to zero for negative *F*^2^. The threshold expression of *F*^2^ > σ(*F*^2^) is used only for calculating *R*-factors(gt) *etc*. and is not relevant to the choice of reflections for refinement. *R*-factors based on *F*^2^ are statistically about twice as large as those based on *F*, and *R*- factors based on ALL data will be even larger.

### Fractional atomic coordinates and isotropic or equivalent isotropic displacement parameters (Å^2^)

**Table:**

	*x*	*y*	*z*	*U*_iso_*/*U*_eq_	
O1	0.24625 (18)	0.49123 (8)	0.59119 (8)	0.0661 (3)	
O2	0.26421 (19)	0.62958 (9)	0.74100 (8)	0.0701 (4)	
H2A	0.2600	0.5770	0.6870	0.105*	
O3	0.24623 (18)	0.81655 (10)	0.75227 (8)	0.0711 (4)	
N1	0.24594 (15)	0.49437 (9)	0.41733 (8)	0.0432 (3)	
H1N	0.2368	0.5389	0.3624	0.052*	
C1	0.23624 (18)	0.54568 (11)	0.50723 (9)	0.0416 (3)	
C2	0.2115 (2)	0.67235 (11)	0.49860 (10)	0.0444 (3)	
H2	0.1931	0.7044	0.4293	0.053*	
C3	0.2122 (2)	0.74646 (11)	0.57694 (11)	0.0463 (3)	
H3	0.1892	0.8224	0.5517	0.056*	
C4	0.2424 (2)	0.73222 (12)	0.69681 (11)	0.0488 (4)	
C5	0.26967 (19)	0.37432 (11)	0.40204 (11)	0.0432 (3)	
C6	0.2264 (2)	0.33549 (12)	0.29220 (11)	0.0502 (4)	
C7	0.2520 (2)	0.21851 (13)	0.27818 (14)	0.0614 (4)	
H7	0.2247	0.1910	0.2060	0.074*	
C8	0.3162 (3)	0.14215 (13)	0.36698 (15)	0.0680 (5)	
H8	0.3303	0.0643	0.3547	0.082*	
C9	0.3591 (3)	0.18185 (13)	0.47378 (14)	0.0648 (4)	
H9	0.4031	0.1307	0.5345	0.078*	
C10	0.3376 (2)	0.29766 (12)	0.49199 (12)	0.0550 (4)	
H10	0.3688	0.3242	0.5650	0.066*	
C11	0.1553 (3)	0.41495 (15)	0.19286 (12)	0.0711 (5)	
H11A	0.1342	0.3721	0.1256	0.107*	
H11B	0.2532	0.4737	0.2041	0.107*	
H11C	0.0326	0.4502	0.1849	0.107*	

### Atomic displacement parameters (Å^2^)

**Table:**

	*U*^11^	*U*^22^	*U*^33^	*U*^12^	*U*^13^	*U*^23^
O1	0.1258 (10)	0.0400 (6)	0.0506 (6)	0.0030 (6)	0.0547 (6)	0.0022 (5)
O2	0.1294 (10)	0.0507 (6)	0.0420 (5)	0.0101 (6)	0.0472 (6)	0.0033 (4)
O3	0.1142 (10)	0.0573 (7)	0.0486 (6)	0.0083 (6)	0.0406 (6)	−0.0134 (5)
N1	0.0616 (7)	0.0386 (6)	0.0353 (5)	−0.0011 (5)	0.0261 (5)	−0.0029 (5)
C1	0.0547 (8)	0.0400 (7)	0.0348 (6)	−0.0020 (6)	0.0233 (6)	−0.0022 (5)
C2	0.0620 (8)	0.0418 (7)	0.0327 (6)	0.0025 (6)	0.0231 (6)	0.0018 (5)
C3	0.0650 (9)	0.0367 (6)	0.0397 (7)	0.0057 (6)	0.0243 (6)	0.0008 (5)
C4	0.0642 (9)	0.0468 (8)	0.0397 (7)	0.0051 (6)	0.0260 (6)	−0.0044 (6)
C5	0.0500 (8)	0.0392 (7)	0.0474 (7)	−0.0045 (5)	0.0272 (6)	−0.0079 (6)
C6	0.0567 (8)	0.0491 (8)	0.0485 (7)	−0.0072 (6)	0.0254 (6)	−0.0151 (6)
C7	0.0701 (10)	0.0541 (9)	0.0626 (9)	−0.0079 (7)	0.0301 (8)	−0.0254 (8)
C8	0.0793 (11)	0.0406 (8)	0.0926 (13)	−0.0042 (7)	0.0441 (10)	−0.0151 (8)
C9	0.0849 (12)	0.0444 (8)	0.0776 (11)	0.0072 (7)	0.0461 (9)	0.0057 (8)
C10	0.0737 (10)	0.0482 (8)	0.0532 (8)	0.0046 (7)	0.0364 (7)	−0.0016 (6)
C11	0.1050 (13)	0.0666 (10)	0.0427 (8)	0.0016 (9)	0.0316 (9)	−0.0134 (7)

### Geometric parameters (Å, °)

**Table:**

O1—C1	1.2355 (14)	C5—C6	1.4005 (17)
O2—C4	1.3015 (17)	C6—C7	1.391 (2)
O2—H2A	0.9200	C6—C11	1.492 (2)
O3—C4	1.2076 (16)	C7—C8	1.373 (2)
N1—C1	1.3387 (14)	C7—H7	0.9300
N1—C5	1.4265 (16)	C8—C9	1.368 (2)
N1—H1N	0.8602	C8—H8	0.9300
C1—C2	1.4779 (18)	C9—C10	1.384 (2)
C2—C3	1.3301 (18)	C9—H9	0.9300
C2—H2	0.9300	C10—H10	0.9300
C3—C4	1.4876 (18)	C11—H11A	0.9600
C3—H3	0.9300	C11—H11B	0.9600
C5—C10	1.3856 (19)	C11—H11C	0.9600
			
C4—O2—H2A	108.1	C7—C6—C11	120.42 (12)
C1—N1—C5	127.52 (10)	C5—C6—C11	122.15 (12)
C1—N1—H1N	116.3	C8—C7—C6	122.44 (14)
C5—N1—H1N	116.2	C8—C7—H7	118.8
O1—C1—N1	122.54 (11)	C6—C7—H7	118.8
O1—C1—C2	122.32 (10)	C9—C8—C7	119.28 (14)
N1—C1—C2	115.14 (10)	C9—C8—H8	120.4
C3—C2—C1	128.49 (11)	C7—C8—H8	120.4
C3—C2—H2	115.8	C8—C9—C10	120.33 (15)
C1—C2—H2	115.8	C8—C9—H9	119.8
C2—C3—C4	132.88 (12)	C10—C9—H9	119.8
C2—C3—H3	113.6	C9—C10—C5	120.33 (13)
C4—C3—H3	113.6	C9—C10—H10	119.8
O3—C4—O2	120.58 (12)	C5—C10—H10	119.8
O3—C4—C3	119.39 (13)	C6—C11—H11A	109.5
O2—C4—C3	120.03 (11)	C6—C11—H11B	109.5
C10—C5—C6	120.17 (12)	H11A—C11—H11B	109.5
C10—C5—N1	122.04 (11)	C6—C11—H11C	109.5
C6—C5—N1	117.77 (11)	H11A—C11—H11C	109.5
C7—C6—C5	117.43 (13)	H11B—C11—H11C	109.5
			
C5—N1—C1—O1	−0.5 (2)	N1—C5—C6—C7	179.30 (12)
C5—N1—C1—C2	179.87 (12)	C10—C5—C6—C11	−179.25 (14)
O1—C1—C2—C3	5.8 (2)	N1—C5—C6—C11	−0.7 (2)
N1—C1—C2—C3	−174.52 (14)	C5—C6—C7—C8	0.3 (2)
C1—C2—C3—C4	2.1 (3)	C11—C6—C7—C8	−179.68 (15)
C2—C3—C4—O3	175.97 (16)	C6—C7—C8—C9	−0.8 (2)
C2—C3—C4—O2	−4.1 (3)	C7—C8—C9—C10	0.2 (2)
C1—N1—C5—C10	−17.3 (2)	C8—C9—C10—C5	0.9 (2)
C1—N1—C5—C6	164.21 (12)	C6—C5—C10—C9	−1.4 (2)
C10—C5—C6—C7	0.8 (2)	N1—C5—C10—C9	−179.82 (13)

### Hydrogen-bond geometry (Å, °)

**Table:**

*D*—H···*A*	*D*—H	H···*A*	*D*···*A*	*D*—H···*A*
N1—H1N···O3^i^	0.86	2.22	3.0665 (14)	167
O2—H2A···O1	0.92	1.56	2.4822 (13)	178

Symmetry codes: (i) *x*, −*y*+3/2, *z*−1/2.
